# Manganese-doped nanotheranostic system for MRI-guided photothermal therapy of malignant pleural mesothelioma: *in vivo* study

**DOI:** 10.3389/fchem.2025.1659283

**Published:** 2025-09-11

**Authors:** Lichang Lei, Hongjiao Zi, ShaoLei Kang, Hui Duan, Zhenghua Zhang, Chao Gao, Ruoyun Xu, Dan Han

**Affiliations:** 1 Department of Medical Imaging, The First Affiliated Hospital of Kunming Medical University, Kunming, China; 2 First Clinical Medical College, Kunming Medical University, Kunming, China

**Keywords:** malignant pleural mesothelioma, photothermal therapy (PTT), magnetic resonance imaging (MRI), polydopamine, manganese(II)

## Abstract

**Introduction:**

Magnetic Resonance Imaging (MRI)-guided photothermal therapy (PTT) holds significant promise for the treatment of solid tumors, however, its diagnostic and therapeutic efficacy in malignant pleural mesothelioma (MPM) remains underexplored. To address the limitations of traditional gadolinium (Gd)-based MRI contrast agents, such as intolerance in patients with renal insufficiency and T1 signal attenuation at high field strengths, as well as oxidative stress damage caused by manganese ion leakage from manganese-based materials, this study aims to develop highly chelated and stable manganese polydopamine (Mn-PDA) multifunctional nanoprobes for MRI monitoring and PTT treatment of MPM.

**Methods:**

Mn^2+^ and dopamine were chelated into Mn-PDA NP nanospheres in an ethanol-water system. The physicochemical properties were characterized using Transmission Electron Microscopy (TEM), Dynamic Light Scattering (DLS), zeta potential, Fourier Transform Infrared Spectroscopy (FT-IR), X-ray Photoelectron Spectroscopy (XPS), and Electron Paramagnetic Resonance (EPR). The photothermal performance under 808 nm laser irradiation was also evaluated. The MRI imaging performance (longitudinal/transverse relaxation rates, r1/r2) and manganese ion deposition quantification of Mn-PDA NPs were evaluated in vitro and in vivo at 3.0 T MR scanner. Additionally, the PTT efficacy of Mn-PDA NPs was evaluated in MSTO-211H cells and xenograft tumor models. Biocompatibility was assessed through H&E staining of major organs and blood biochemical analyses.

**Results:**

Mn-PDA NPs exhibited a spherical morphology with uniform size (~200 nm), along with high photothermal conversion efficiency, favorable T1-weighted MRI contrast enhancement and low cytotoxicity. At 8 hours post-injection in vivo, Mn-PDA NPs resulted in a 42.9% enhancement in T1-weighted MRI signals within MPM tumors, accompanied by specific tumor accumulation. Under 808 nm laser irradiation, Mn-PDA NPs inhibited MPM tumor growth, as evidenced by reduced tumor volume, expanded areas of tumor necrosis (H&E staining), and downregulated Ki-67 expression. Moreover, stable body weight, normal histological features of major organs, and unaltered blood biochemical parameters were observed in the animals.

**Discussion:**

These findings indicate that Mn-PDA NPs are effective dual-functional agents, serving as both MRI contrast agents and photothermal therapeutics. They exhibit good tumor targeting ability, excellent imaging performance, potent therapeutic efficacy, and high biosafety, thereby offering a promising therapeutic strategy for MPM management.

## 1 Introduction

Malignant pleural mesothelioma (MPM), a highly aggressive cancer associated with asbestos exposure, has shown an increasing global incidence (Huang et al., 2023). Its insidious onset and prolonged latency (20–40 years) mean over 70% of patients present with unresectable disease, necessitating systemic therapy. However, the first-line cisplatin-pemetrexed regimen offers limited benefit (Castagneto et al., 2008). Recent immunotherapies provide alternatives for refractory disease (Cedres and Felip, 2022), but profound interpatient heterogeneity results in lower efficacy compared to malignancies like melanoma or renal cell carcinoma. Combination strategies, such as cisplatin with bevacizumab (Zalcman et al., 2016) or pembrolizumab plus chemotherapy (Chu et al., 2023) yield modest improvements, especially for non-epithelioid MPM. Nevertheless, median overall survival remains ∼18 months, with a 5-year survival rate of only 10% (Febres-Aldana et al., 2024), underscoring the urgent need for novel, comprehensive diagnostic and therapeutic approaches.

Photothermal therapy (PTT) nanoplatforms offer promising non-invasive and precise targeting for solid tumors, particularly superficial ones (Overchuk et al., 2023), yet their application in MPM remains underexplored. PTT employs photothermal conversion agents (PTCAs) to transform light energy into localized heat for tumor ablation. Diverse PTCAs, including metal-based nanomaterials (Moreira et al., 2025), carbon nanomaterials (Xing et al., 2025) and sulfides (Wu et al., 2024), demonstrate efficacy under near-infrared (NIR) light. However, most inorganic PTCAs suffer from poor biodegradability, raising long-term safety concerns. Furthermore, conventional high-temperature PTT (>50°C) risks damaging healthy tissue and inducing inflammation (Rybinski et al., 2013), while low-temperature PTT (<42°C) often triggers thermoresistance via upregulated heat shock proteins (HSPs) (Tang et al., 2024). MRI, with its non-invasiveness, absence of ionizing radiation, superior soft-tissue contrast, and high spatial resolution (Jin et al., 2021), is ideally suited for crucial high-quality tumor imaging within PTT workflows, enabling monitoring, precise guidance, and efficacy/off-target assessment. To develop an MRI-trackable, biodegradable photothermal nanoplatform, polydopamine (PDA) nanoparticles were introduced to chelate paramagnetic ions such as Fe(III), Mn(II), or Gd(III) (Jia et al., 2024; Wang et al., 2017; Zhao et al., 2025), enhancing MRI contrast and mitigating metal-induced oxidative stress (Yang et al., 2023). While Gd(III) is the most widely used MRI contrast agent, it poses risks like nephrogenic systemic fibrosis (NSF) (Telgmann et al., 2012) and shows reduced T1 efficacy at high magnetic fields (Jiang et al., 2023). In contrast, Mn, as an essential trace element, provides strong T1 contrast and activates the cGAS-STING pathway to enhance tumor immunity (Du et al., 2025), making it an ideal alternative.

In recent years, researchers have explored the integration of manganese ions with polydopamine (PDA) through various methods to enhance MRI-guided photothermal therapy. Notable approaches include the synthesis of core-shell nanostructures by layering PDA and manganese oxide (MnO_2_) onto hollow mesoporous silica nanoparticles (HS) (Shi et al., 2023), as well as the development of mesoporous polydopamine nanoparticles (MPDAPs) loaded with manganese ions (Wu et al., 2021). Some studies have also involved embedding Mn^2+^ into cross-linked PDA coatings on rod-like cellulose nanocrystals (Shen et al., 2022). However, these research efforts have not fully leveraged magnetic resonance quantitative tracking technology to accurately determine the distribution and metabolism of Mn^2+^. Furthermore, whether adsorbed into the voids of mesoporous PDA nanoparticles or incorporated into hollow mesoporous silica nanoparticles (HS), manganese ions face challenges of leakage and unintended accumulation in normal tissues, especially causing neurological toxicity (Li et al., 2021). This issue stems from the unstable binding between the carrier and manganese ions, highlighting the need for more robust methods to ensure controlled drug delivery and minimize off-target effects. To address these limitations, we developed a novel nanotheranostic probe designed to guide PTT for MPM under high-frequency MRI monitoring. This probe employs PDA chelated withMn^2+^ in an optimal ratio to create size-controlled nanoparticles. Furthermore, the enhanced EPR effect promotes the accumulation of nanoparticles in MPM tumors, while the acidic tumor microenvironment (TME) of MPM facilitates the degradation of Mn-PDA nanoparticles (NPs), accelerating the release of Mn^2+^. Through systematic *in vitro* and *in vivo* evaluations, we demonstrated the potential of this probe for integrated diagnosis and treatment of MPM.

## 2 Materials and methods

### 2.1 Materials

Dopamine hydrochloride (PDA) and Manganese (II) sulfate monohydrate (MnSO_4_·H_2_O) were sourced from Aladdin Reagent (Shanghai, China). Tris-(hydroxymethyl)-aminomethane (Tris-HCL) and dimethyl sulfoxide (DMSO) were obtained from Sigma-Aldrich (Missouri, United States). The CCK-8 reagent was sourced from Dojindo Laboratories (Japan), and Calcein AM/PI Cell Staining Kit was supplied by Soleibio (Shanghai, China). All reagents were analytically pure and used without further modification. Distilled water was used throughout the experiments.

### 2.2 Mn-PDA NPs synthesis

Dopamine hydrochloride (90 mg) and manganese (II) sulfate monohydrate (3 mg) were suspended in deionized water and stirred continuously for 24 h. Subsequently, this pre-formed chelate solution (0.6 mL) was introduced into a water/ethanol mixture (6 mL each) under ultrasonication. The pH of the mixture was adjusted to 8.5–9 using Tris buffer and mixed continuously for 6 h. The resulting Mn-PDA NPs were recovered by centrifugation and washed sequentially with ethanol and water twice.

### 2.3 Particle characterization

Size distribution and zeta potential were determined by Malvern laser diffraction analysis. Transmission electron microscopy (TEM, JEM-2100) revealed morphology. Chemical bond changes were analyzed using Fourier-transform infrared spectroscopy (FT-IR, Thermo Fisher Scientific). Manganese oxidation states were determined using X-ray photoelectron spectroscopy (XPS) and electron paramagnetic resonance (EPR). The manganese concentration was measured using inductively coupled plasma atomic emission spectrometry (ICP-AES, Agilent 5100). To monitor the release of manganese ions from Mn-PDA NPs, they were placed in pH 7.4 and pH 6.0 PBS buffer solutions with GSH at 10 mM respectively, and stirred in a 37°C water bath. Samples were taken at fixed time points (0, 2, 4, 6, 8, 10, 12, 24, 48, 72 h) and the manganese ion content was analyzed using ICP-AES.

### 2.4 In vitro photothermal experiment

To evaluate photothermal performance, the suspensions of deionized water (H_2_O), DOPA NPs, and Mn-PDA NPs were exposed to an 808 nm laser (1 W/cm^2^, 5 min) while monitoring temperature at 30-s intervals. Separately, we assessed the dose-dependent response by irradiating Mn-PDA NPs at concentrations of 0, 50, 100, and 200 μg/mL under identical laser conditions for 10 min. Thermal stability was evaluated by subjecting Mn-PDA NPs to four consecutive laser irradiation cycles (808 nm, 1 W/cm^2^), recording temperature throughout.

### 2.5 *In vitro* MR imaging

For phantom studies, solutions of Mn-PDA NPs were prepared at varying Mn(II) concentrations (0, 0.01, 0.03, 0.06, 0.13, 0.25 mM). Imaging was performed on a 3.0T MRI scanner (GE Signa Architect) using T1-weighted (T1WI), T2-weighted (T2WI), and MAGiC sequences, quantitative T1map and T2map were derived from the MAGiC data (Zhao et al., 2025). Specific scanning parameters are listed in Table 1. Regions of interest (ROIs) were consistently positioned across samples at the same level to measure longitudinal and transverse relaxation times (T1 and T2). Then longitudinal and transverse relaxivity (r_1_ and r_2_) were determined from linear regressions correlating nanoparticle concentration with reciprocal relaxation times.

**TABLE 1:** MRI scan parameters.

Images	T1WI	T2WI	MAGiC
pulse sequence	FSE	SE	MAGiC
TE (ms)	15	100	15/106
TR (ms)	500	2000	5,000
FOV(mm^2^)	50 × 50	50 × 50	80 × 80
Matrix	256 × 256	256 × 256	256 × 256
Spacing (mm)	0	0	0
ST (mm)	1.5	1.5	2
Voxel	0.2	0.3	0.5
Bandwidth (kHz)	32	25	20.8

Note: T1WI, T1-weighted imaging; T2WI, T2-weighted imaging; TE, echo time; TR, time of repetition; FOV, field of view; Spacing, Interslice Spacing; ST, slice thickness.

### 2.6 *In vitro* cell experiment

#### 2.6.1 Cell culture

In this study, the immortalized mesothelial cell line MET-5A and the human biphasic MPM cell line MSTO-211H were procured from the Cell Bank of the Chinese Academy of Sciences. MET-5A cells were cultured in Procel’s specific complete medium and MSTO-211H cells were maintained in RPMI 1640 culture medium enriched with 10% FBS and 100 U/mL penicillin-streptomycin. All cells were maintained at 37°C under a 5% CO_2_ atmosphere.

#### 2.6.2 Cell viability assay

MSTO-211H and MET-5A cells were plated into Corning^®^ 96-well plates and incubated for 12 h. The medium was refreshed, and cells were treated with Mn-PDA NPs at concentrations of 1, 10, 50, 100, 200, and 400 μg/mL for 24 h. For photothermal experiments, cells were incubated with saline, DOPA NPs, or Mn-PDA NPs for 4 h, then exposed to an 808 nm laser (1 W/cm^2^) for 5 min. Following irradiation, the cells were maintained in culture for another 4 h. After treatment, the medium was removed, and each well was replaced with 100 μL of fresh culture medium containing 10 μL of CCK-8 solution. Following incubation for 1 h and gently shaking for 5 min, the optical density (OD) at 450 nm was determined via a microplate reader. The cell survival rate was assessed based on Equation 1.

$$Cell\;survival\;rate=\frac{{OD}_{sample}-{OD}_{blank}}{{\text{OD}}_{control}-{OD}_{blank}}\times 100\%$$ (1)

#### 2.6.3 Calcein-AM/PI-based cell viability assay

MSTO-211H cells were incubated with saline, DOPA NPs, or Mn-PDA NPs for 4 h, while the laser irradiation group was exposed to laser light for 5 min (1 W/cm^2^) and subsequently incubated for an additional hour. Following PBS washes, cells were stained following the Calcein-AM/PI double staining kit instructions and incubated at 37°C for an additional 30 min. Subsequent fluorescence imaging at 488 nm and 543 nm was performed with an inverted fluorescence microscope.

### 2.7 *In vivo* animal experiments

#### 2.7.1 Modeling MPM Xenografts in nude mice

Female BALB/c nude mice, aged 4–6 weeks (certificate no.: SCXK (Dian) K2020-004; Kunming Medical University Animal Experiment Center) were utilized in this study. Animal care and experimental procedures complied with the ethical standards sanctioned by the Kunming Medical University Animal Ethics and Welfare Committee (Approval Number: kmmu20241769). For tumor cell implantation, 1.0 × 10^6^MSTO-211H cells suspended in 50 μL PBS were mixed with an equal volume (50 μL) of Matrigel. This cell-Matrigel mixture (100 μL) was then injected subcutaneously into the right axilla of each mice. Tumor progression was monitored via measurements of body weight and tumor volume every 2 days. Treatment administration and MRI acquisition were initiated once the tumor volume tumor reaches 150 mm^3^.

#### 2.7.2 *In vivo* MRI evaluation

To evaluate contrast enhancement within the tumor region, MRI was performed. The mice were administered an IV dose of 150 μL Mn-PDA NPs solution (10 mg/kg) and were scanned afterward using a 3.0 T MRI system (GE Medical Systems) with a 50 mm mouse coil (Shanghai Chenguang Medical Technologies, China) at 0, 4, 8, 12, and 24 h post-injection. T1-weighted (T1WI) and T2-weighted (T2WI) images were obtained to assess tumor location, shape, and dimensions. The MAGiC sequence was utilized for the quantitative evaluation of T1 and T2 relaxation parameters. Define the region of interest (ROI) in the tumor center and adjacent areas, avoiding necrotic regions, with two attending physicians independently analyzing the images three times. Increase rate of T1 value (ΔT1) was calculated as follows: ΔT1 = [T1 value (unenhanced) - T1 value (enhanced)]/T1 value (unenhanced) ×100% . 8 h post-administration of saline (control) and Mn-PDA NPs, the mice were euthanized, and major organs (heart, liver, spleen, lung, kidney, and tumor) were assessed using ICP-AES to evaluate manganese ion distribution.

#### 2.7.3 *In vivo* treatment evaluation

Thirty-six tumor-bearing mice were randomly divided into four experimental groups, designated as follows: 1) saline control (Saline), 2) saline with laser irradiation (Saline + L), (3) DOPA NPs with laser irradiation (DOPA NPs + L), and (4) Mn-PDA NPs with laser irradiation (Mn-PDA NPs + L). After intravenous administration of the drug (150 μL, 10 mg/kg), mice in the laser irradiation group received laser irradiation (1 W/cm^2^) at the tumor site for 5 min, 8 h post-injection. Tumor temperature was monitored in real-time using a thermal imager. This treatment protocol was administered every 2 days over a 14-day period. Body weight and tumor volume were monitored at 48-h intervals, with tumor volume calculated using the formula: volume = (1/2) × a^2^ × b (where a corresponds to the tumor’s width and b reflects its length). At the 24-day mark, MRI scans were performed on mice from the respective treatment groups, scanning parameters consistent with those detailed in Section 2.7.2 (*In vivo* MRI evaluation). Additionally, tumor tissues were stained with H&E and Ki-67 to assess treatment efficacy, whereas major organs were stained with H&E to evaluate biosafety.

### 2.8 Statistical analysis

Statistical analyses were conducted using Origin software (version 2021) and GraphPad Prism (version 10.1.1). Independent two-tailed t-tests were used to analyze the differences between two groups, while one-way analysis of variance (ANOVA) was employed to evaluate the differences among multiple groups. Data were collected in triplicate and presented as mean ± standard deviation (mean ± SD). Differences were established as statistically significant at the following thresholds of significance: p < 0.05 (∗), p < 0.01 (∗∗), p < 0.001 (∗∗∗), and p < 0.0001 (∗∗∗∗).

## 3 Results and discussion

### 3.1 Preparation and characterization of the nanotherapeutic probe

**FIGURE 1:**
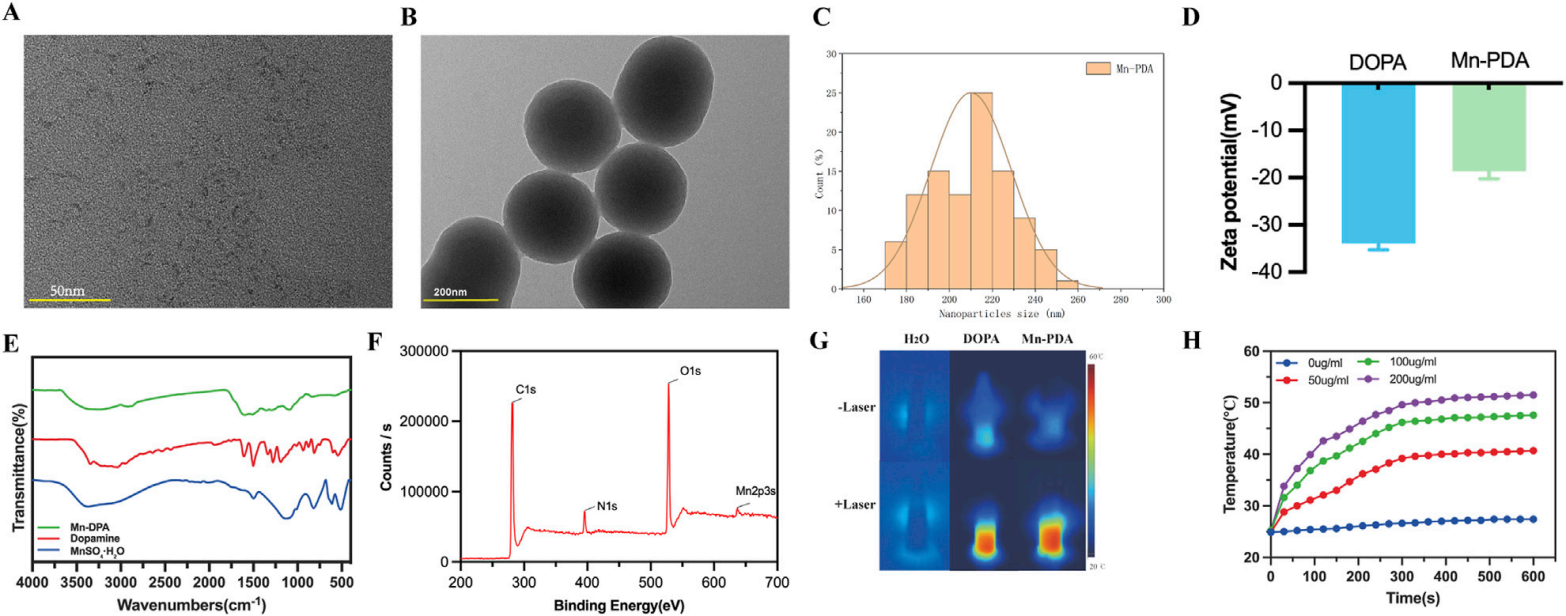
Characterization of Mn-PDA NPs. **(A)** TEM image of Mn-PDA chelating complex. Scale bar: 50 nm. **(B)** TEM image of Mn-PDA nanospheres. Scale bar: 200 nm. **(C)** Hydrodynamic size distribution of Mn-PDA NPs. **(D)** Zeta potential of Mn-PDA NPs. **(E)** FT-IR spectra of MnSO_4_·H_2_O (blue), dopamine (red), and Mn-PDA NPs (green). **(F)** X-ray photoelectron spectroscopy (XPS) of Mn-PDA NPs. **(G)** Infrared thermal images of water (H_2_O), DOPA NPs, and Mn-PDA NPs under 808 nm laser irradiation (1 W/cm^2^). **(H)** Temperature curves of Mn-PDA NPs at varying concentrations (808 nm irradiation, 1 W/cm^2^).

Mn-PDA NPs were synthesized based on previously reported methods (Zhang et al., 2023), and the average diameter and zeta potential values of the NPs were obtained by the Dynamic Light Scattering (DLS). As shown in Figure 1A, TEM demonstrates that Mn^2+^ ions initially chelate with PDA to form particulate complexes, exhibiting a uniform size distribution centered at ∼2 nm. Subsequently, under alkaline conditions, these complexes undergo spontaneous oxidative polymerization to form homogeneous nanospheres with an average diameter of 210.3 ± 18.4 nm (PDI = 0.18; Figures 1B,C). The zeta potential of DOPA was −33.9 ± 1.1 mV. After chelating manganese ions, the zeta potential of Mn-PDA NPs increased to −18.7 ± 1.2 mV (Figure 1D),We speculate in a DOPA-rich system, carboxyl groups dominate the surface charge. Despite Mn ^2+^ coordination and protonated amines, the high density of -COO- ensures an overall negative potential. The FT-IR spectra (Figure 1E) provide critical evidence for Mn^2+^ chelation: (i) Broadening and reduction in intensity of the N-H (∼3,350 cm^−1^) and C=C peaks (blue-shifted to 1,630 cm^−1^); (ii) Disappearance of SO_4_^2−^ signatures (1,110 cm^−1^); (iii) Emergence of new Mn-O bonds (560 cm^−1^), confirming successful coordination between Mn^2+^ and catechol groups. Constituent elements of Mn-PDA NPs was examined by XPS revealed peaks at 285, 400, 531, and 637 eV, corresponding to C1s, N1s, O1s, and Mn2p3s, respectively (Figure 1F). As shown in Supplementary Figure S1C,D, the EPR spectrum of Mn-PDA NPs exhibits a characteristic six-line hyperfine splitting pattern (centered at g = 2.0090), indicative of isolated Mn^2+^ (3d^5^, S = 5/2) in a high-symmetry ligand field. In contrast, the EPR spectrum of crystalline MnSO_4_·H_2_O (Supplementary Figure S1A,B) displays only broad peaks due to pronounced ZFS anisotropy induced by low-symmetry distortion, which obscures the hyperfine splitting. Furthermore, the molar concentration of Mn(II) in Mn PDA NPs was determined via ICP-AES as 5.8%, confirming the successful preparation of Mn-PDA NPs.As shown in Supplementary Figure S2, manganese ion (Mn^2+^) release was minimal in the pH 7.4 environment, remaining below approximately 0.5 μg/mL. In contrast, under conditions of pH 6.0 and 10 mM GSH, the Mn^2+^ release increased significantly by 6 h and stabilized at levels between 2 and 4 μg/mL, even after 72 h, the cumulative release accounted for only about 5% of the total potential. The results demonstrated that the release of Mn^2+^ from Mn-PDA NPs was minimal under the tested conditions, indicating the excellent stability of the nanoparticles *in vitro*.

PDA can chelate various metal ions, such as Mn^2+^ and Fe^3+^, and absorb and convert near-infrared (NIR) light into heat energy. Previous studies have demonstrated that Mn^2+^ coordination in PDA significantly enhances photothermal conversion efficiency (η > 45%), enabling effective tumor ablation at reduced laser doses (Wu et al., 2021). As shown in Figure 1G, a series of concentrations of the nanoparticles in an aqueous solution were irradiated with an 808 nm laser (1 W/cm^2^, 5 min), with ultrapure water used as a control. Minimal temperature change was observed in water under laser irradiation. In contrast, the temperature change in Mn-PDA NPs aqueous solutions occurred in a concentration-dependent manner, with the maximum temperature reaching 48.5°C (8.0°C higher than that in DOPA NPs at 100 μg/mL). Furthermore, as the Mn-PDA NPs concentration increased, the temperature rose gradually (Figure 1H).

### 3.2 *In vitro* MR imaging and cell cytotoxicity

**FIGURE 2:**
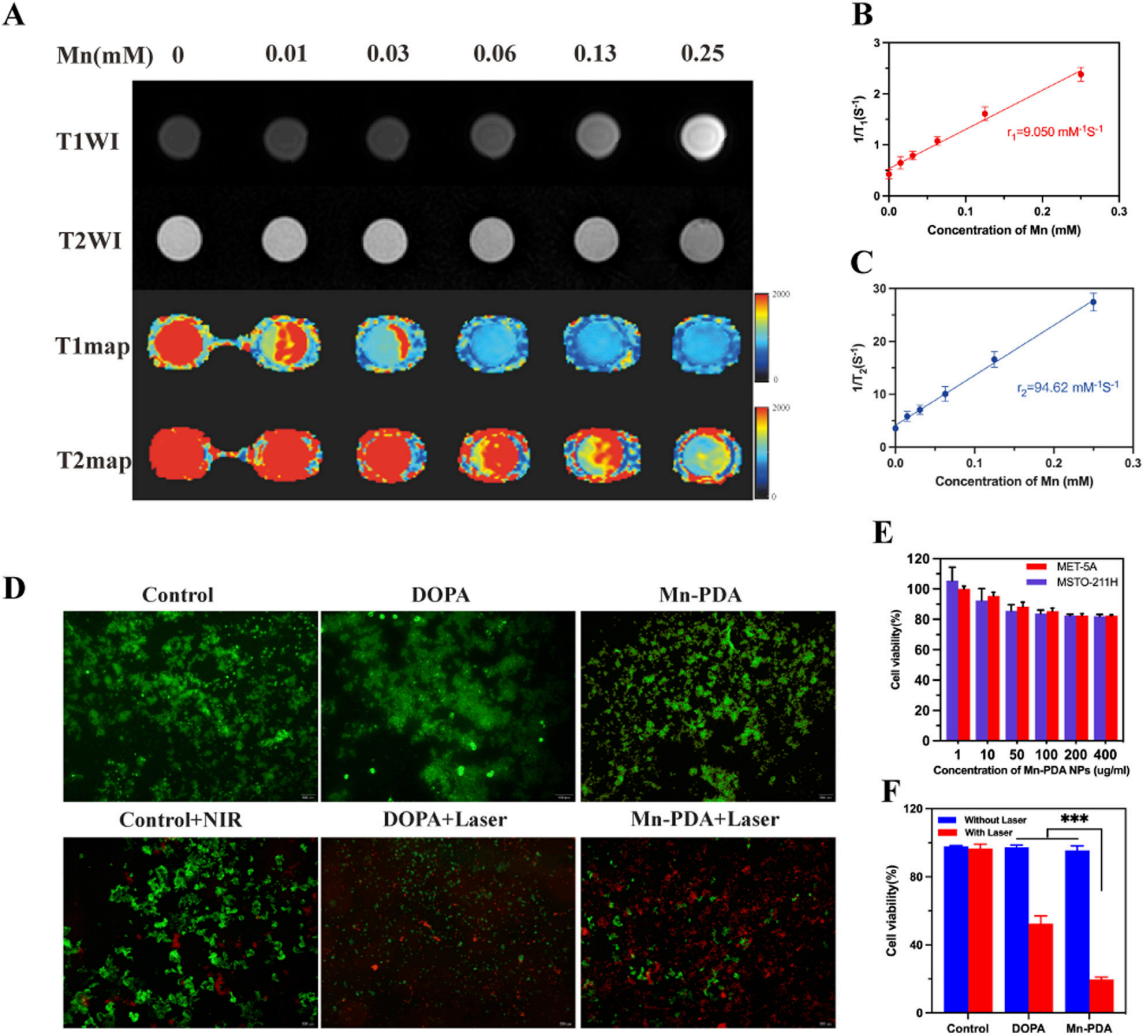
MR images and Cellular cytotoxicity of the nanoparticles. **(A)** T1WI, T2WI, T1map and T2map MRI images of Mn-PDA NPs with different concentrations at 3T field strength. **(B)** Corresponding Longitudinal relaxation rates (r_1_, s^−1^mM^−1^) and **(C)** transverse relaxation rates (r_2_, s^−1^mM^−1^) of Mn-PDA NPs. **(D)** Live/dead fluorescence staining (Calcein-AM/PI) of MSTO-211H cells post-treatments (Scale bar: 200 μm). **(E)** Viability of MeT-5A and MSTO-211H cells after 4 h incubation with Mn-PDA NPs. **(F)** Viability of MSTO-211H cells treated with DOPA, Mn-PDA NPs (±808 nm laser irradiation, 1 W/cm^2^, n = 3).

Manganese-based nanoparticles, leveraging their inherent paramagnetism, have emerged as promising T1-MRI contrast agents, offering an alternative to Gd-DTPA. Recent studies highlight the synergistic effects between Mn^2+^ and the PDA matrix in enhancing proton spin relaxation (Ma et al., 2024). To evaluate Mn-PDA NPs for MRI-guided PTT in tumor theranostics, their MR performance was assessed using a 3.0 T MR scanner. As shown in Figure 2A, Mn-PDA NPs exhibited a concentration-dependent brightening effect on T1-MR images and a darker effect on T2-MR images. T1map revealed a longitudinal relaxation rate (r1) of 9.05 mM^−1^s^−1^ (Figure 2B), nearly double that of Gd-DTPA (r1 = 3.5–5.6 mM^−1^s^−1^ at 3.0 T) (Wahsner et al., 2019), and a transverse relaxation rate (r2) of 94.62 mM^−1^s^−1^ (Figure 2C), slightly lower than SPIO NPs (r2 = 118.2 mM^−1^s^−1^) (Choi et al., 2012), yet still meeting clinical standards.

Biocompatibility is crucial in therapeutic applications, so the cytotoxicity of Mn-PDA NPs was evaluated in 2 cell lines (MeT-5A and MSTO-211H) using the CCK-8 assay. Even at a concentration as high as 400 μg/mL, the viability of both cell lines exceeded 80.0%, demonstrating favorable biocompatibility (Figure 2E). Furthermore, the *in vitro* therapeutic effects of DOPA NPs and Mn-PDA NPs were evaluated by labeling MSTO-211H cells with Calcein-AM/PI and CCK-8 assay. In Figure 2D, the majority of MSTO-211H cells exhibited high viability in the absence of NIR irradiation, as indicated by intense green fluorescence. However, under 808-nm NIR irradiation (1 W/cm^2^, 5 min), MSTO-211H cells treated with Mn-PDA NPs showed significant damage, with cell viability dropping to 19.7% ± 1.1% (from 95.5% ± 2.4% in non-irradiated controls) and a marked increase in red fluorescence. In contrast, cells treated with DOPA NPs exhibited only minimal signs of apoptosis, with viability moderately reduced to 52.5% ± 1.2% (from 97.2% ± 3.7% of controls) (Figure 2F).In conclusion,the Mn-PDA-mediated phototherapy could result in significant combined antitumor efficacy of Mn-PDA NPs against MPM.

### 3.3 *In vivo* MR imaging

**FIGURE 3:**
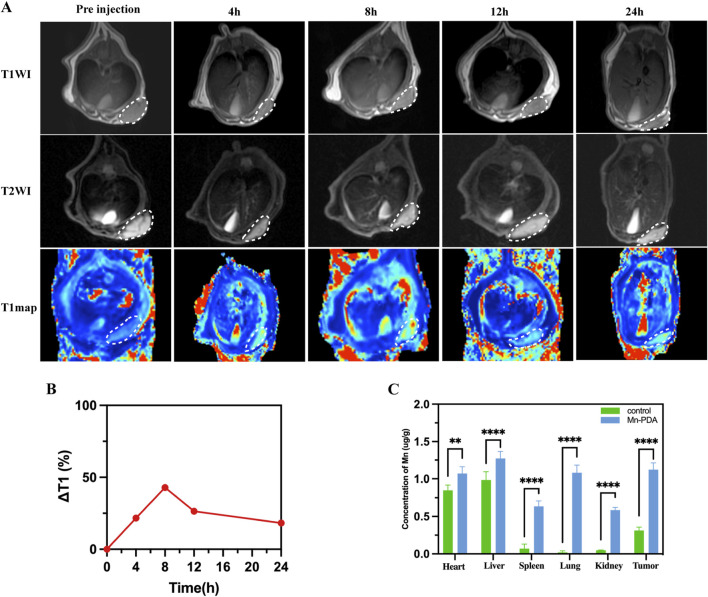
*In vivo* imaging effect of the nanoprobes in mice. **(A)** *In vivo* T1WI,T2WI and T1map MRI images of MSTO-211H tumor-bearing mice receiving an intravenous tail injection of Mn-PDA NPs. **(B)** ΔT1 of tumor from mice at different times post-injection of Mn-PDA NPs. **(C)** Mean manganese concentrations in major organs and tumors of tumor-bearing mice via ICP-AES at 8 h post-injection of Mn-PDA NPs and saline (control).

Given the advantageous characteristics of Mn-PDA NPs, we conducting *in vivo* MRI studies in MPM tumor-bearing BALB/c nude mice. Following intravenous injection, 3.0 T MRI scans revealed time-dependent signal changes at the tumor site over 24 h. T2-weighted signal slightly decreased, while T1-weighted signal progressively intensified, peaking at 8 h post-injection (Figure 3A). The corresponding longitudinal relaxation time (T1) decreased significantly from 1,293 ± 62.6 ms (pre-injection) to 737 ± 82.6 ms at 8 h post-injection. This peak enhancement, likely influenced by Mn-PDA NP biodegradation, represented a 42.9% ± 4.1% reduction in T1, corresponding to an increase in T1 -weighted signal intensity. Although the signal intensity declined thereafter, the T 1 value remained elevated at 24 h (1,058 ± 59.6 ms), which was 18.2% ± 1.3% lower than the pre-injection T1 values. This confirmed efficient tumor accumulation and sustained MRI contrast enhancement by the Mn-PDA NPs (Figure 3B).

The Mn^2+^ concentration in the tumor was quantified by ICP-AES analysis at 8 h post-injection. The control group administered with saline had a concentration of 0.31 ± 0.03 mg/kg, whereas the Mn-PDA NPs group showed a significantly higher level of 1.12 ± 0.07 mg/kg (Figure 3C). Mn-PDA NPs are shown to be an effective and reliable tumor MR imaging agent with enhanced imaging capabilities. Compared to the control group, Mn^2+^ accumulation was observed in other organs, including the heart (1.07 ± 0.09 mg/kg), liver (1.27 ± 0.11 mg/kg), spleen (0.64 ± 0.05 mg/kg), lung (1.08 ± 0.1 mg/kg), and kidney (0.59 ± 0.03 mg/kg). This distribution is consistent with the expected biological pattern for non-targeted nanoparticles eliminated via phagocytosis.

### 3.4 *In vivo* antitumor efficacy

**FIGURE 4:**
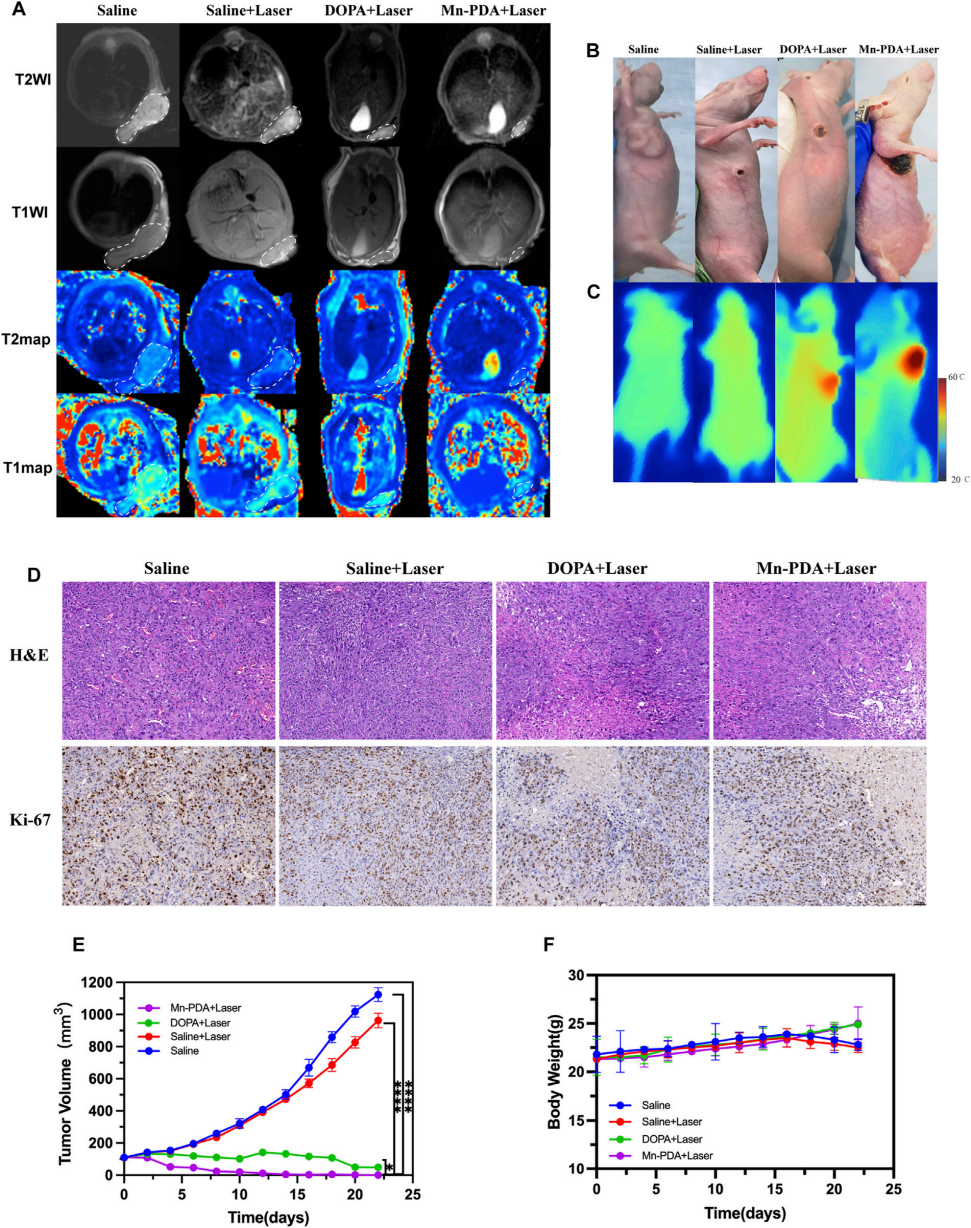
*In vivo* Therapeutic efficacy and biocompatibility of Mn-PDA NPs. The representative axial MR images of mice from different groups, white dashed box indicate tumor regions **(A)**. Representative photographs **(B)** and *in vivo* infrared thermographs **(C)** of mice post-injection of Saline, DOPA NPs and Mn-PDA NPs under 808 nm laser irradiation (1W/cm^2^, 5 min). **(D)** H&E staining and KI-67 IHC of tumor images after treatment (Scale bar: 50 μm. n = 5). **(E)** Tumor volumes of mice for different treatments. **(F)** Bodyweight changes of mice after various treatments (n = 5,*P < 0.05, **P < 0.01, ***P < 0.001).

The *in vivo* anti-tumor efficacy of Mn PDA NPs combined with PTT in the MSTO-211H mice modelwas evaluated. According to the MR imaging results, laser irradiation was performed 8 h after tail vein injection. As shown in Figure 4C, after 5 min of 808 nm laser exposure, the saline group showed only a slightly temperature increase (26.4 °C–32.4°C; ΔT ≈6.0 °C), the DOPA NPs group reached 42.5 °C which potentially insufficient for complete ablation due to tumor cell thermotolerance mediated by heat shock proteins (Hsps), while the Mn-PDA NPs group experienced a significant rise and rapidly reached 48.9°C (ΔT ≈22.4°C), confirming potent photothermal conversion and self-targeting capability. In addition, MR imaging provided critical information for treatment optimization by identifying tumor location, nanoparticle distribution, and monitoring peritumoral reactions such as exudation and edema (Jiang et al., 2022). As shown in Figures 4A–C, the MnPDA NPs + L group demonstrated the best therapeutic effect. Specifically, Figure 4B revealed significant tumor growth inhibition, with tumors appearing almost completely resolved while preserving the surrounding normal tissues. However, MRI images (Figure 4A) still revealed trace amounts of residual tumor tissue within the deep subcutaneous layers and the chest wall musculature. This finding underscores the distinct advantage of magnetic resonance imaging in detecting and monitoring residual disease to accurately assess the effectiveness of tumor treatment. Histological analysis (H&E and Ki-67 staining) of excised tumors further confirmed the therapeutic effects. Tumor cells in the saline and saline + L groups maintained close packing and normal morphology, whereas clear apoptotic features, including pyknotic nuclei and tissue vacuolation, were observed in both the DOPA NPs + L and Mn-PDA NPs + L groups. Moreover, Ki-67 staining corroborated these findings, showing markedly reduced expression levels and proliferation in Mn-PDA NPs + L groups compared to controls (Figure 4D).

Therapeutic efficacy of Mn-PDA NPs was evaluated in the MPM tumor model. Mice treated with Mn-PDA NPs + L, DOPA NPs + L, or saline (controls)+ L showed contrasting outcomes: treatment groups exhibited steady weight gain alongside tumor regression, whereas controls experienced weight loss concomitant with uncontrolled tumor growth. Critically, Mn-PDA NPs + L demonstrated the most potent tumor inhibition, recapitulating *in vitro* findings and confirming superior efficacy of photothermal therapy (Figures 4E,F).

**FIGURE 5:**
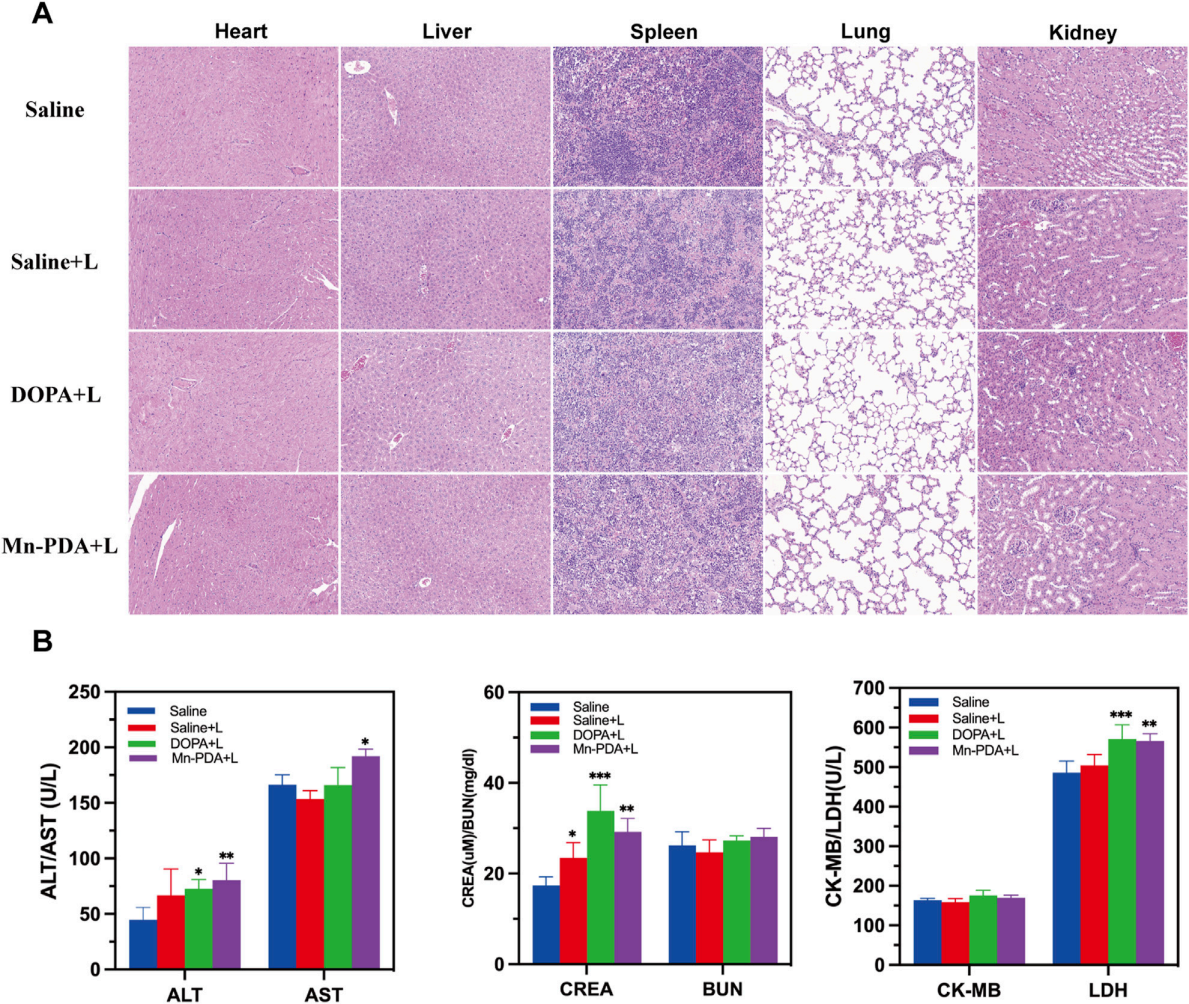
Biosafety of Major Organs after Mn-PDA NPs Treatment. **(A)** H&E staining of major organs (heart, liver, spleen, lung, kidney) after treatment (scale bar: 50 μm, n = 5). **(B)** Blood biochemical parameters analysis of major organs (heart, liver, spleen, lung, kidney) after treatment (n = 5, *P < 0.05, **P < 0.01, ***P < 0.001).

Given the therapeutic efficacy of Mn-PDA NPs + L, its potential organ toxicity was assessed. H&E staining of major organs (heart, liver, spleen, lung, kidney) revealed no pathological abnormalities in any group (Figure 5A). As shown in Figure 5B, the hematological indices, including liver-associated enzymes (AST, ALT), renal function indicators (CRE, BUN), and cardiac biomarkers (CK-MB, LDH), all stayed within normal reference ranges across all experimental groups, suggesting minimal toxicity effects in the tumor-bearing mice. Collectively, Mn-PDA NPs demonstrated good biocompatibility and potent tumor suppression. However, their clinical translation is delayed due to potential toxicity risks, especially the accumulation of free Mn^2^+ in dopaminergic neurons, which may lead to neurotoxicity. Although some clinical trials (Zhang et al., 2023) and approved agents, like Mn-DPDP (Hamm, et al., 1992), suggest short-term safety ofmanganese-based nanocomposites, there is a lack of long-term toxicological data and animal models that closely mimic humans. Further research is needed to confirm their safety and effectiveness for clinical use.

## 4 Conclusion

Our study developed a biomimetic nanoplatform Mn-PDA NPs, with significant T1 MRI characteristics (r_1_ = 9.05 mM^−1^s^−1^) and a mean diameter of approximately 200 nm, designed for MRI-guided photothermal therapy of biphasic MPM. The T1 MRI signal of Mn PDA NPs reached its peak 8 h after intravenous administration, enabling precise prediction of nanoparticle accumulation in biphasic MPM tumors. When exposed to 808 nm laser irradiation, the nanoplatform generated mild photothermal effects, successfully suppressing tumor growth in biphasic MPM models without inducing significant systemic toxicity. These findings underscore the potential of an integrated diagnostic and therapeutic nanosystem for treating biphasic MPM. However, this study has certain limitations, and future research should focus on optimizing nanoparticle delivery efficiency and monitoring their metabolism and degradation in complex *in vivo* environments to further enhance therapeutic efficacy and safety.

## Data Availability

The original contributions presented in the study are included in the article/Supplementary Material, further inquiries can be directed to the corresponding author.

## Supplementary material

SUPPLEMENTARY FIGURE S1 EPR spectra of MnSO_4_·H_2_O **(A, B)** and Mn-PDA NPs **(C,D)**.

SUPPLEMENTARY FIGURE S2 In Vitro Mn^2+^ Release from Mn-PDA NPs.

## References

[B1] Castagneto B. Botta M. Aitini E. Spigno F. Degiovanni D. Alabiso O. (2008). Phase II study of pemetrexed in combination with carboplatin in patients with malignant pleural mesothelioma (MPM). Ann. Oncol. 19 (2), 370–373. 10.1093/annonc/mdm501 18156144

[B2] Cedres S. Felip E. (2022). 3-Year CheckMate743 outcomes: ringing in immunotherapy for the treatment of malignant pleural mesothelioma. Ann. Oncol. 33 (5), 457–459. 10.1016/j.annonc.2022.03.004 35306158

[B3] Choi W. I. Kim J. Y. Heo S. U. Jeong Y. Y. Kim Y. H. Tae G. (2012). The effect of mechanical properties of iron oxide nanoparticle-loaded functional nano-carrier on tumor targeting and imaging. J. Control Release 162 (2), 267–275. 10.1016/j.jconrel.2012.07.020 22824783

[B4] Chu Q. Perrone F. Greillier L. Tu W. Piccirillo M. C. Grosso F. (2023). Pembrolizumab plus chemotherapy *versus* chemotherapy in untreated advanced pleural mesothelioma in Canada, Italy, and France: a phase 3, open-label, randomised controlled trial. Lancet 402 (10419), 2295–2306. 10.1016/S0140-6736(23)01613-6 37931632

[B5] Du Y. Mai Y. Liu Z. Lin G. Luo S. Guo C. (2025). Synergistic provoking of pyroptosis and STING pathway by multifunctional manganese-polydopamine nano-immunomodulator for enhanced renal cell carcinoma immunotherapy. Adv. Healthc. Mater, e2500141. 10.1002/adhm.202500141 40394938

[B6] Febres-Aldana C. A. Fanaroff R. Offin M. Zauderer M. G. Sauter J. L. Yang S. R. (2024). Diffuse pleural mesothelioma: advances in molecular pathogenesis, diagnosis, and treatment. Annu. Rev. Pathol. 19, 11–42. 10.1146/annurev-pathol-042420-092719 37722697

[B7] Hamm B. Vogl T. J. Branding G. Schnell B. Taupitz M. Wolf K. J. (1992). Focal liver lesions: MR imaging with Mn-DPDP--initial clinical results in 40 patients. Radiology 182 (1), 167–174. 10.1148/radiology.182.1.1309218 1309218

[B8] Huang J. Chan S. C. Pang W. S. Chow S. H. Lok V. Zhang L. (2023). Global incidence, risk factors, and temporal trends of mesothelioma: a population-based study. J. Thorac. Oncol. 18 (6), 792–802. 10.1016/j.jtho.2023.01.095 36775192

[B9] Jia Y. Gao F. Wang P. Bai S. Li H. Li J. (2024). Supramolecular assembly of Polydopamine@Fe nanoparticles with near-infrared light-accelerated cascade catalysis applied for synergistic photothermal-enhanced chemodynamic therapy. J. Colloid Interface Sci. 676, 626–635. 10.1016/j.jcis.2024.07.089 39053410

[B10] Jiang G. Fan D. Tian J. Xiang Z. Fang Q. (2022). Self-Confirming magnetosomes for tumor-targeted T1/T2 dual-mode MRI and MRI-Guided photothermal therapy. Adv. Healthc. Mater 11 (14), e2200841. 10.1002/adhm.202200841 35579102

[B11] Jiang Q. Xu H. Zhang W. Wang Y. Xia J. Chen Z. (2023). Mn(II)-hemoporfin-based metal-organic frameworks as a theranostic nanoplatform for MRI-guided sonodynamic therapy. Biomater. Sci. 11 (24), 7838–7844. 10.1039/d3bm01316b 37889225

[B12] Jin X. Yang W. Xu Y. Bian K. Zhang B. (2021). Emerging strategies of activatable MR imaging probes and their advantages for biomedical applications. VIEW-CHINA 2 (5), 20200141. 10.1002/viw.20200141

[B13] Li J. Deng Y. Peng D. Zhao L. Fang Y. Zhu X. (2021). Sodium P-aminosalicylic acid attenuates manganese-induced neuroinflammation in BV2 microglia by modulating NF-κB pathway. Biol. Trace Elem. Res. 199 (12), 4688–4699. 10.1007/s12011-021-02581-w 33447908

[B14] Ma G. Zhang X. Zhao K. Zhang S. Ren K. Mu M. (2024). Polydopamine nanostructure-enhanced water interaction with pH-Responsive manganese sulfide nanoclusters for tumor magnetic resonance contrast enhancement and synergistic ferroptosis-photothermal therapy. ACS Nano 18 (4), 3369–3381. 10.1021/acsnano.3c10249 38251846

[B15] Moreira A. F. Filipe H. A. L. Miguel S. P. Ribeiro M. J. Coutinho P. (2025). Recent advances in smart gold nanoparticles for photothermal therapy. Nanomedicine (Lond) 20 (11), 1339–1353. 10.1080/17435889.2025.2500912 40329458 PMC12140457

[B16] Overchuk M. Weersink R. A. Wilson B. C. Zheng G. (2023). Photodynamic and photothermal therapies: synergy opportunities for nanomedicine. ACS Nano 17 (9), 7979–8003. 10.1021/acsnano.3c00891 37129253 PMC10173698

[B17] Rybinski M. Szymanska Z. Lasota S. Gambin A. (2013). Modelling the efficacy of hyperthermia treatment. J. R. Soc. Interface 10 (88), 20130527. 10.1098/rsif.2013.0527 23985732 PMC3785818

[B18] Shen Y. Li X. Huang H. Lan Y. Gan L. Huang J. (2022). Embedding Mn2+ in polymer coating on rod-like cellulose nanocrystal to integrate MRI and photothermal function. Carbohydr. Polym. 297, 120061. 10.1016/j.carbpol.2022.12006 36184155

[B19] Shi Y. Zhou M. Zhang Y. Wang Y. Cheng J. (2023). MRI-guided dual-responsive anti-tumor nanostructures for synergistic chemo-photothermal therapy and chemodynamic therapy. Acta Biomater. 158, 571–582. 10.1016/j.actbio.2022.12.053 36586501

[B20] Tang Z. Liu Y. Ni D. Wang H. Zhang H. Liu Y. (2024). Near-infrared light-responsive polymeric micelles for enhanced photothermal-chemodynamic therapy. Biomater. Sci. 12 (11), 3333–3345. 10.1039/d4bm00350a

[B21] Telgmann L. Wehe C. A. Künnemeyer J. Bülter A. C. Sperling M. Karst U. (2012). Speciation of Gd-based MRI contrast agents and potential products of transmetalation with iron ions or parenteral iron supplements. Anal. Bioanal. Chem. 404 (8), 2133–2141. 10.1007/s00216-012-6404-x 23001305

[B22] Wahsner J. Gale E. M. Rodríguez-Rodríguez A. Caravan P. (2019). Chemistry of MRI contrast agents: current challenges and new frontiers. Chem. Rev. 119 (2), 957–1057. 10.1021/acs.chemrev.8b00363 30350585 PMC6516866

[B23] Wang Z. Carniato F. Xie Y. Huang Y. Li Y. He S. (2017). High relaxivity gadolinium-polydopamine nanoparticles. Small 13 (43). 10.1002/smll.201701830 29024478

[B24] Wu Y. Huang Y. Tu C. Wu F. Tong G. Su Y. (2021). A mesoporous polydopamine nanoparticle enables highly efficient manganese encapsulation for enhanced MRI-guided photothermal therapy. Nanoscale 13 (13), 6439–6446. 10.1039/d1nr00957e 33885524

[B25] Wu D. Huang Q. Sha S. Xue F. Huang G. Tian Q. (2024). Engineering of copper sulfide mediated by phototherapy performance. Wiley Interdiscip. Rev. Nanomed Nanobiotechnol 16 (1), e1932. 10.1002/wnan.1932 37853634

[B26] Xing Y. Jing R. Kang J. Li Y. Zhang H. Tang X. (2025). Carbon-based nanomaterials in photothermal therapy guided by photoacoustic imaging: state of knowledge and recent advances. Curr. Med. Chem. 32 (2), 238–257. 10.2174/0109298673287448240311112523 38529603

[B27] Yang X. Chen Y. Guo J. Li J. Zhang P. Yang H. (2023). Polydopamine nanoparticles targeting ferroptosis mitigate intervertebral disc degeneration *via* reactive oxygen Species depletion, iron ions chelation, and GPX4 ubiquitination suppression. Adv. Sci. (Weinh) 10 (13), e2207216. 10.1002/advs.202207216 36951540 PMC10161035

[B28] Zalcman G. Mazieres J. Margery J. Greillier L. Audigier-Valette C. Moro-Sibilot D. (2016). Bevacizumab for newly diagnosed pleural mesothelioma in the Mesothelioma Avastin cisplatin Pemetrexed Study (MAPS): a randomised, controlled, open-label, phase 3 trial. Lancet 387 (10026), 1405–1414. 10.1016/S0140-6736(15)01238-6 26719230

[B29] Zhang K. Qi C. Cai K. (2023). Manganese-Based Tumor immunotherapy. *Adv. Mat.* May 35 (19), e2205409. 10.1002/adma.202205409 36121368

[B30] Zhang R. Liu M. Liu S. Liang X. Lu R. Shuai X. (2023). Holmium (III)-doped multifunctional nanotheranostic agent for ultra-high-field magnetic resonance imaging-guided chemo-photothermal tumor therapy. Acta Biomater. 172, 454–465. 10.1016/j.actbio.2023.10.017 37863345

[B31] Zhao J. Li M. Wu Q. Qiao G. Jiang H. Zhao S. (2025). Targeted reprogramming of TREM2-Positive macrophages and MRI-Guided immune monitoring in colorectal cancer using CSF1R@Mn@MPDA-Antitrem2 nanoplatform. Adv. Healthc. Mater 14 (18), e2501027. 10.1002/adhm.202501027 40417930

[B32] Zhao X. B. Zhao H. Du W. J. Zhang H. (2025). Quantitative MRI reveals infrapatellar fat pad changes after running a marathon. PeerJ 13, e19123. 10.7717/peerj.19123 40124601 PMC11930216

