# Supplementary figures and images for: Manganese-doped nanotheranostic system for MRI-guided photothermal therapy of malignant pleural mesothelioma: in vivo study
Source: Front Chem. 2025 Sep 11;13:1659283. doi: 10.3389/fchem.2025.1659283 (PMC12461733; doi:10.3389/fchem.2025.1659283)

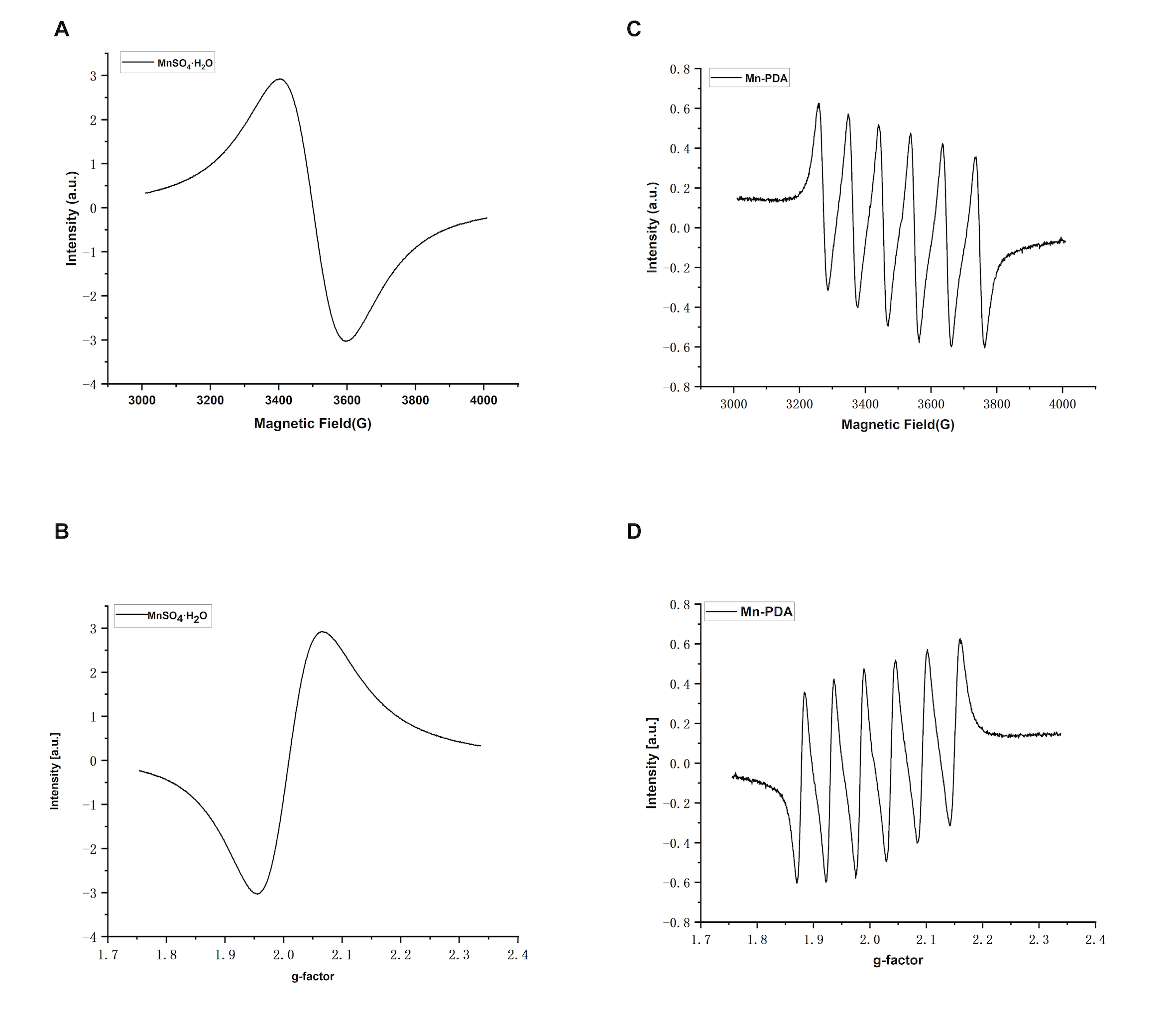

Supplement: Supplementary file 1 [file Supplementaryfile1.tif]

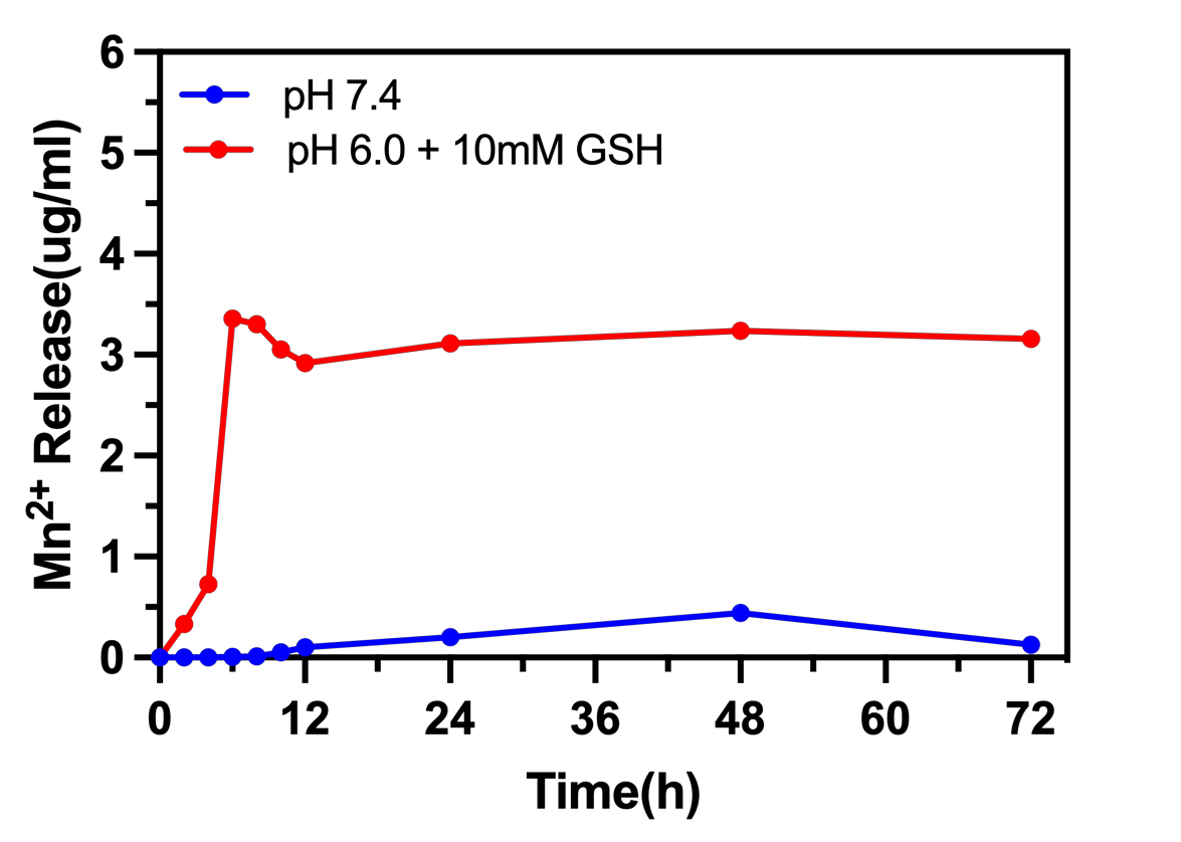

Supplement: Supplementary file 2 [file Supplementaryfile2.tiff]
